# Genetic Signatures of Demographic Changes in an Avian Top Predator during the Last Century: Bottlenecks and Expansions of the Eurasian Eagle Owl in the Iberian Peninsula

**DOI:** 10.1371/journal.pone.0133954

**Published:** 2015-07-31

**Authors:** Eva Graciá, Joaquín Ortego, José Antonio Godoy, Juan Manuel Pérez-García, Guillermo Blanco, María del Mar Delgado, Vincenzo Penteriani, Irene Almodóvar, Francisco Botella, José Antonio Sánchez-Zapata

**Affiliations:** 1 Ecology Area, Department of Applied Biology, Miguel Hernández University, Elche, Spain; 2 Genetic and Cultural Biodiversity Group, Hunting Resources Research Institute, CSIC-UCLM-JCCM, Ciudad Real, Spain; 3 Department of Integrative Ecology, Doñana Biological Station, CSIC, Seville, Spain; 4 Department of Evolutionary Ecology, National Museum of Natural History, CSIC, Madrid, Spain; 5 Department of Biosciences, Metapopulation Research Group, University of Helsinki, Helsinki, Finland; 6 Research Unit of Biodiversity, UMIB, UO-CSIC-PA, Oviedo University, Campus de Mieres, Mieres, Spain; 7 Department of Conservation Biology, Doñana Biological Station, CSIC, Seville, Spain; Instituto de Higiene e Medicina Tropical, PORTUGAL

## Abstract

The study of the demographic history of species can help to understand the negative impact of recent population declines in organisms of conservation concern. Here, we use neutral molecular markers to explore the genetic consequences of the recent population decline and posterior recovery of the Eurasian eagle owl (*Bubo bubo*) in the Iberian Peninsula. During the last century, the species was the object of extermination programs, suffering direct persecution by hunters until the 70’s. Moreover, during the last decades the eagle owl was severely impacted by increased mortality due to electrocution and the decline of its main prey species, the European rabbit (*Oryctolagus cuniculus*). In recent times, the decrease of direct persecution and the implementation of some conservation schemes have allowed the species’ demographic recovery. Yet, it remains unknown to which extent the past population decline and the later expansion have influenced the current species’ pattern of genetic diversity. We used eight microsatellite markers to genotype 235 eagle owls from ten Spanish subpopulations and analyse the presence of genetic signatures attributable to the recent population fluctuations experienced by the species. We found moderate levels of differentiation among the studied subpopulations and Bayesian analyses revealed the existence of three genetic clusters that grouped subpopulations from central, south-western and south-eastern Spain. The observed genetic structure could have resulted from recent human-induced population fragmentation, a patchy distribution of prey populations and/or the philopatric behaviour and habitat selection of the species. We detected an old population bottleneck, which occurred approximately 10,000 years ago, and significant signatures of recent demographic expansions. However, we did not find genetic signatures for a recent bottleneck, which may indicate that population declines were not severe enough to leave detectable signals on the species genetic makeup or that such signals have been eroded by the rapid demographic recovery experienced by the species in recent years.

## Introduction

Animal populations may be reduced and isolated by both natural and anthropogenic factors. Habitat fragmentation by means of the transformation of natural and semi-natural habitat may lead to a patchy distribution of populations and communities and it is considered as a major paradigm in conservation biology [1]. Fragmentation processes may generate many different landscape patterns that share some consequences for animal populations, including reduced population size and increased isolation [2]. Furthermore the effects of fragmentation might be reinforced by changes in habitat quality (e.g. variation in food resources) of the remnant patches [3]. However, populations might also be fragmented by means of direct human persecution, an important driver of biodiversity loss worldwide that affects particularly large long-lived organisms such as top predators [4, 5]. Among them, raptors have long been persecuted as a result of a human-wildlife conflict, in which raptors were thought to limit game prey [6, 7]. The majority of species were killed extensively in Europe until the implementation of national and international legislation for their protection, which allowed the recovery of many of them along the last decades [6].

The populations of Eurasian eagle owls (*Bubo bubo*) in Europe experienced such dynamics in their recent history. This owl is the largest strigiform in the world and it is widely distributed in the Palearctic [8, 9], inhabiting a variety of habitats from boreal coniferous and deciduous forests to Mediterranean scrub, steppes and deserts [8, 10]. Direct persecution led to the extinction of this species in large areas of Europe, such as northern Germany in 1830, the Netherlands in the late 19th century, Luxembourg in 1903, Belgium in 1943, central and western Germany in the 1960s [11, 12] and in some areas of Spain in the 1950s-70s [13]. In the Iberian Peninsula, human persecution of predators peaked by the mid of 20th century with the financial support of public institutions that paid for killing raptors, corvids and carnivores [14]. In Spain the eagle owl was among the species most affected by direct human persecution but also by the decline of its preferred prey, the European rabbit (*Oryctolagus cuniculus*) [13]. The populations of this keystone prey species crashed as consequence of the outbreak of two viral diseases, myxomatosis in the 1950s and rabbit haemorrhagic disease in the late 1980s, a fact that also strongly affected the populations of many other top predators from the Iberian Peninsula [15]. In turn, the decline of rabbit populations further enhanced predator persecution by humans [16]. Although rabbit populations did not totally recover, in the 1970s most top predators and particularly raptors were protected by law, allowing their recovery [14, 17]. Thus, Spanish eagle owl populations face a recent range expansion associated with lower human-induced mortality rates and the local progressive recovery of its main prey.

In this work, we explore the genetic consequences of the recent population decline and the posterior recovery experienced by the eagle owl in the Iberian Peninsula. We expected to find genetic signatures attributable to the above described demographic fluctuations undergone by this species during the last century. In particular, during some generations after demographic contractions, random changes in allele frequencies, caused by dominant genetic drift, are expected to result in genetic patterns that reflect departures from stability situations: i) low levels of genetic diversity and increased differentiation among isolated populations [18]; ii) significant departures from Hardy-Weinberg equilibrium conditions and linkage disequilibrium among loci [19]; and iii) an excess of heterozygosity at selectively neutral loci as a consequence of the preferential loss of low frequency alleles [20]. When these processes are accompanied by population fragmentation, the combination of low effective population sizes and reduced dispersal can promote differentiation and subpopulations may become genetically structured [21–23]. Contrarily, the preponderance of gene flow during demographic expansion phases promotes: i) genetic homogenization of subpopulations [24] and ii) heterozygosity deficits and high proportions of rare alleles [25]. Under further stability situations, isolation by distance patterns could also appear if a balance between genetic drift and gene flow is achieved [21, 22]. However, it remains poorly understood the impact that rapid subsequent demographic changes may have had on the neutral genetic patterns, or how the particular characteristics of organisms could condition the transition from unstable and stable situations. Here, we use microsatellite markers to analyze the potential genetic consequences associated with the recent demographic fluctuations experienced by the eagle owl in the Iberian Peninsula. In particular, we first analyze the patterns of genetic structure in order to understand whether population fragmentation has resulted in detectable genetic subdivision. Second, we employ neutral markers and different analytical approaches to test for bottlenecks and expansions and understand the genetic consequences of the demographic fluctuations experienced by the species. Finally, we use microsatellite data to estimate contemporary effective population sizes and evaluate the conservation status of the species.

## Materials and Methods

### Ethical Standards

Eagle owl subpopulations were sampled under the authorization of Junta de Andalucía-Consejería de Medio Ambiente (permits nos.: SCFFSAFR/GGG RS-260/02 and SCFFS-AFR/CMM RS-1904/02), Generalitat Valenciana-Consellería de Territorio i Habitatge (with express authorization of J. Jiménez as head of the Wildlife Department), Junta de Comunidades de Castilla-La Mancha (permits nos.: 36456, 308003, 284189, 361269), Comunidad de Madrid-Consejería de Medio Ambiente, Vivienda y Ordenación del Territorio (permits nos.: 10/072240.9/13), Junta de Castilla y León-Consejería de Fomento y Medio Ambiente (permits nos.: EP/CyL/282/13). All authorisations covered the blood withdrawal method used to sample the chicks. A. Urmeneta provided samples from Bárdenas Reales with authorization of Gobierno de Navarra (as responsible of Reserva de la Biosfera de Bárdenas Reales). We also obtained the authorization of owners for entry in the private lands in which we worked. When this study was performed it was not yet mandatory in Spain to get permission from an ethics committee (legislation: Real Decreto 223/1988). Anyway, the capture and manipulation of chicks posed little risk to the birds, which were returned immediately to their nests.

### Study area, sampling and laboratory procedures

**Fig 1:**
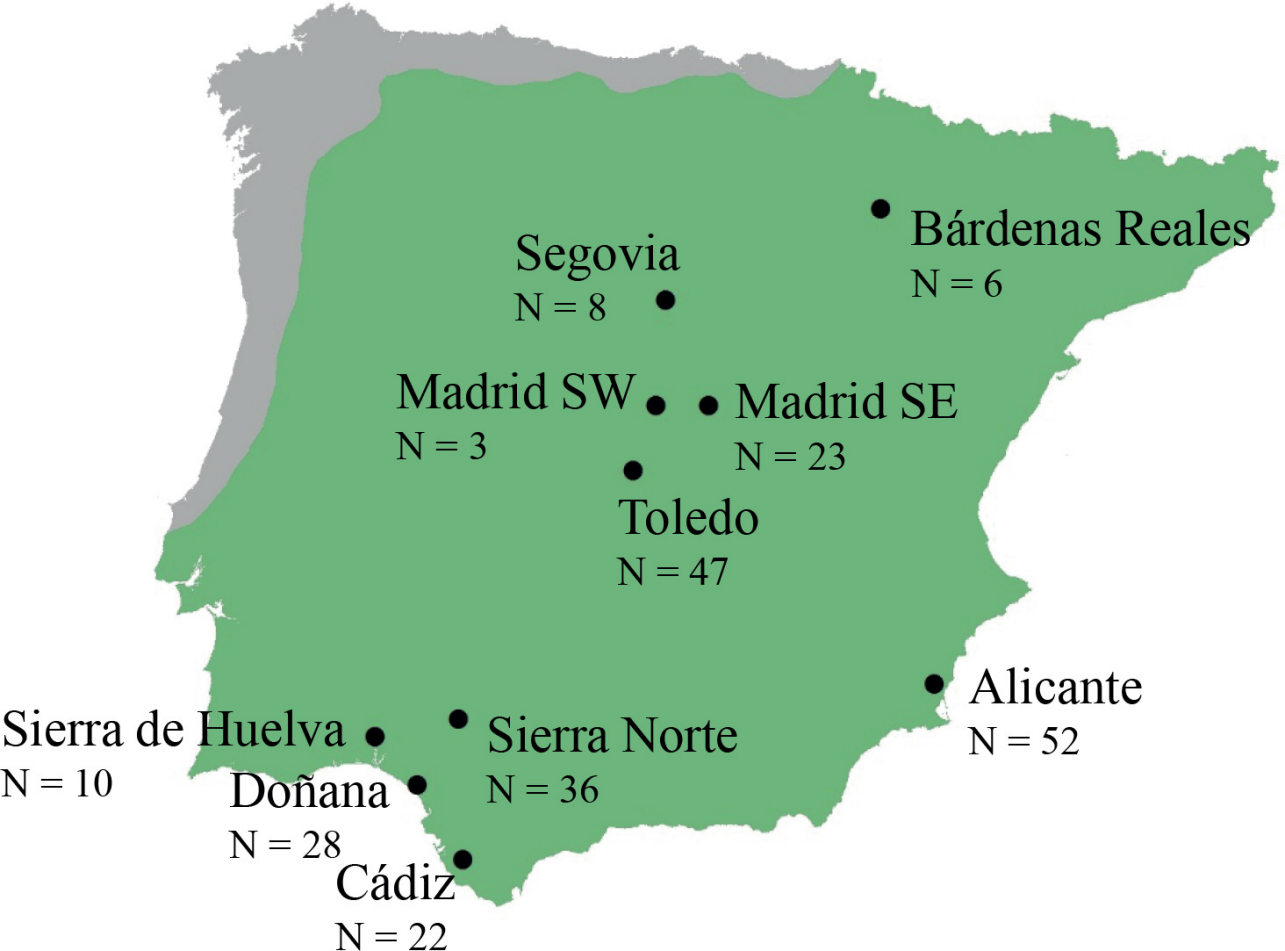
Distribution of the Eurasian eagle owl (*Bubo bubo*) in the Iberian Peninsula. This species can be considered continuous across the Iberian Peninsula, with the exception of the coastal region in the center and north of Portugal, and Galicia and the Cantabrian coasts in Spain. The number of analysed chicks (N) in each subpopulation is indicated.

From 2004 to 2012, we monitored 253 eagle owl territories from ten subpopulations in the Iberian Peninsula. We sampled one chick per nest, with the exception of Bardenas Reales site, where 6 chicks from 4 nests were sampled. Eurasian eagle owl distribution can be considered continuous across the Iberian Peninsula [26], therefore we defined subpopulations as clusters of territories located in the same geographical region (Fig 1). For spatial analyses, we used centroid coordinates calculated from all nest sampled within each subpopulation.

Blood samples were collected from the brachial vein when chicks were 20–25 days old as recommended by Penteriani et al. [27] and preserved in 70% ethanol until DNA extraction. For genomic DNA isolation we followed the salt-extraction protocol described by Salah et al. [28]. A subset of 19 samples were used for the screening of 15 microsatellite markers developed for the Eurasian eagle owl [29], the spotted owl (*Strix occidentalis lucida*) [30], and the Lanyu scops owl (*Otus elegans botelensis*) [31]. We discarded from further analyses five loci that resulted monomorphic (Bb145, Bb111, Bb101, 1C6, 8G11), and other two due to their low rate of amplification in some populations (Bb42, Bb131). The final set comprised eight microsatellite markers: Bb126, Bb101, 15A6, Oe2-57, Oe3-7, Oe045, Oe054, and Oe128 (S1 Table). PCR reactions consisted of 2μl of 10x NH_4_ Buffer, 0.8μl of 50 mM MgCl_2_, 0.8 μl of 10 mM solution of each primer, 0.05 μl of 100mM dNTPs Mix, 0.08 μl of Taq (Bioline), and 12.17 μl of distilled water. PCR cycling conditions included a denaturalization step for 30s at 94°C, followed by 35 cycles of 30s at 92°C, 30s at the annealing temperature (S1 Table) and 30 s at 72°C, with a final 10 min extension at 72°C. Forward primers were labelled with fluorescent dyes (S1 Table). PCR products were visualized by electrophoresis in 1.5% agarose gels and later run on an ABI 3100 analyzer (Applied Biosystems) for allele scoring using GeneMapper 4.0 software (Applied Biosystems).

### Genetic diversity and structure analyses

Genotypic linkage disequilibrium (LD) between each pair of microsatellite loci was tested by subpopulation using the Markov chain Monte Carlo (MCMC) method implemented in Genepop 4.0 [32]. All loci were examined for null alleles using Micro-Checker 2.2.3 [33]. We used GenAlEx 6.5.01 [34] to assess departures from Hardy-Weinberg equilibrium (HWE) of loci and calculate descriptive statistics of genetic diversity for each locality with more than six sampled individuals: average number of alleles (*N*_A_), mean number of effective alleles (*A*_E_), average observed heterozygosity (*H*_O_), unbiased average expected heterozygosity (u*H*_E_), and inbreeding coefficient (*F*_IS_).

We analyzed spatial patterns of genetic structure using two Bayesian clustering methods, Structure 2.3.4 [35–37] and Geneland 4.0.3 [38, 39], which identify groups (*K*) that maximize HWE and minimize LD within them. First, we ran Structure from *K* = 1 to *K* = 11, without prior population information, using the admixture model and the option ‘correlated allele frequencies’. The burn-in was set to 10,000 and the number of iterations to 100,000. To evaluate the convergence and estimate the optimal genetic clustering, we ran 10 replicates for each value of *K*. We also ran Structure considering the origin of individuals as prior information (“Locprior” option), which increases the sensibility of Structure in clustering populations with low genetic divergence [37]. The number of populations best fitting the data set was defined both using log probabilities [Pr(X|*K*)] [35] and the Δ*K* method [40], as implemented in Structure Harvester [41]. The second clustering method, Geneland, has some similarities with Structure but uses the spatial distribution of samples as prior information and deals with loci with potential null alleles. We ran the MCMC five times from *K* = 1 to *K* = 11, with the following parameters: 100,000 MCMC iterations, a thinning of 100, uncorrelated allele frequency model, null allele model, maximum rate of Poisson process fixed to 100, uncertainty attached to spatial coordinates fixed to 10 km, maximum number of nuclei in the Poisson-Voronoi tessellation fixed to 300 and a Dirichlet model for allelic frequencies as suggested in Guillot et al. [39]. We inferred the optimal value of *K* with these first five runs and ran the software five additional times with *K* fixed to the selected value but using correlated allele frequencies due to its highest power to detect subtle genetic patterns when the optimal *K* value is known [39]. The posterior probability of population membership was computed for these 5 runs after a burn-in of the first 10% of the saved iterations. Structure results were visualized using Distruct [42].

In order to further explore the spatial pattern of genetic differentiation, we used GenAlEx to calculate pairwise *F*_ST_ and *F*’_ST_ values between subpopulations. We used Mantel tests to analyse the relationship between the linearized estimates of pairwise *F*_ST_ and *F*’_ST_ and the logarithm of geographical distances between pairs of populations [43]. Moreover, we employed hierarchical AMOVAs to determine how genetic variation is distributed among regions, populations, individuals or within individuals, when considering the genetic groups previously inferred in the Bayesian clustering analyses above described. Madrid SW and Bardenas Reales were not included in these analyses due to their low sample size and the presence of siblings in the sample, respectively. Finally, we estimated the effective population sizes (*Ne*) of groups using the LD method implemented in Neestimator 1.3 [44]. We calculated *Ne* estimates assuming monogamy, excluding all allele frequencies lower than 0.05, and selecting a parametric procedure to construct 95% confidence intervals.

### Inference of past demographic processes

The genetic signatures of the recent population bottlenecks and expansions of the Eurasian eagle owl in the Iberian Peninsula were explored using the software Bottleneck 1.2.02 [45]. We tested the excess or defect of heterozygosity that is likely to arise from a population size reduction or expansion, respectively [20]. We assumed the stepwise mutation model (SMM) and the two-phase mutation model (TPM) with 78% stepwise mutations (*p*_*s*_), 22% multi-step mutations (*p*_*g*_) and a variance of 3.1 among multiple steps, as recommended for microsatellite data [45]. The significance of the analyses was assessed with one-tailed Wilcoxon signed-rank tests based on 1,000 replications. Each analysis was independently carried out for each inferred genetic cluster. Moreover, to prevent errors or biases due to the effect of potential null alleles, each analysis was repeated after discarding those loci showing a significant homozygote excess.

Demographic declines were also assessed for the inferred clusters using the M-ratio test as implemented in M_p_val [46]. This analysis is based on the ratio of the total number of alleles (*k*) over the range of allele sizes (*r*) since it is expected that during a bottleneck the random loss of alleles will result in a reduced M-ratio (M = *k*/*r*). To determine the significance of the reduction of M, the observed value is compared to a distribution of values obtained from simulated populations considering a hypothetical pre-bottleneck *Ne*, the mutation rates of loci (μ), the proportion of multi-step mutations (*p*_*g*_) and the mean size of multi-repeat mutations (*Δ*_*g*_). Critical_M software was used to calculate critical values (M_c_) set at the lower 5% tail of the distribution below which it can be assumed that the population has experienced a significant reduction in size [46]. We calculated θ (θ = 4*Ne*μ), considering a mutation rate (μ) of 5x10^-4^ (as recommended by Garza and Williamson [46]) and different values of pre-bottleneck Ne: 5, 500, 1000, 3000, 6000 and 25000. In consequence, θ ranged from 0.01 to 50. For the remaining two TPM parameters, we took as a reference for our analyses the recently reported average of *p*_*g*_ = 22% and *Δ*_*g*_ = 3.1, calculated among 15 vertebrate species [47].

Finally, we used Msvar 1.3 [48, 49] to infer changes in the effective size of the whole population. This Bayesian coalescent method is based on the observed allele distributions and allele frequencies and assumes that a stable population with a determinate size (*N*_1_) started to decrease (or increase) linearly or exponentially some time ago (*T*) towards the current population size (*N*_0_). Based on this assumption and using the MCMC approach the method not only allows the estimation of *N*_0_ and *N*_1_, but also *T*. Mutation rates (θ *=* 2*N*_0_μ) were assumed under the SMM model and, as for the rest of the parameters, we considered wide uninformative priors under a lognormal distribution. The generation time of Eurasian eagle owl was set at 4 years, the average age of the reproductive population (V. Penteriani and M.M. Delgado, unpublished data). Because we tested both recent or ancient expansion and bottleneck processes, we ran the program five independent times under the exponential growth model, using different hyperprior means for *T* and *N*_0_, to represent stable, decreasing or increasing demographic histories at different times (S2 Table). The total number of iterations in each analysis was 1 × 10^8^, with a thinning interval of 2.5 × 10^4^ iterations. We discarded the first 10% of total iterations to avoid bias in parameter estimation due to starting conditions. The distribution of the remaining data was plotted against prior distributions to see the consistence of the results over the different runs and used to obtain the lower (10%), the median (50%) and upper quantile (90%) of the posterior distributions.

## Results

### Spatial patterns of genetic diversity and structure

We detected a total of 91 alleles, ranging from 4 to 24 at Bb101 and Oe2 loci, respectively. We did not find significant LD for any of the 28 pair-wise locus combinations at subpopulations. When all individuals were pooled, Micro-Checker indicated that null alleles may be present by detecting a significant heterozygote deficiency at five out of the eight employed markers, possibly as a consequence of cryptic population structure (i.e. Wahlund effect, see the next paragraph) (S1 Table). However, GenAlEx revealed that no marker consistently deviated from HWE across the analysed subpopulations.

**Fig 2:**
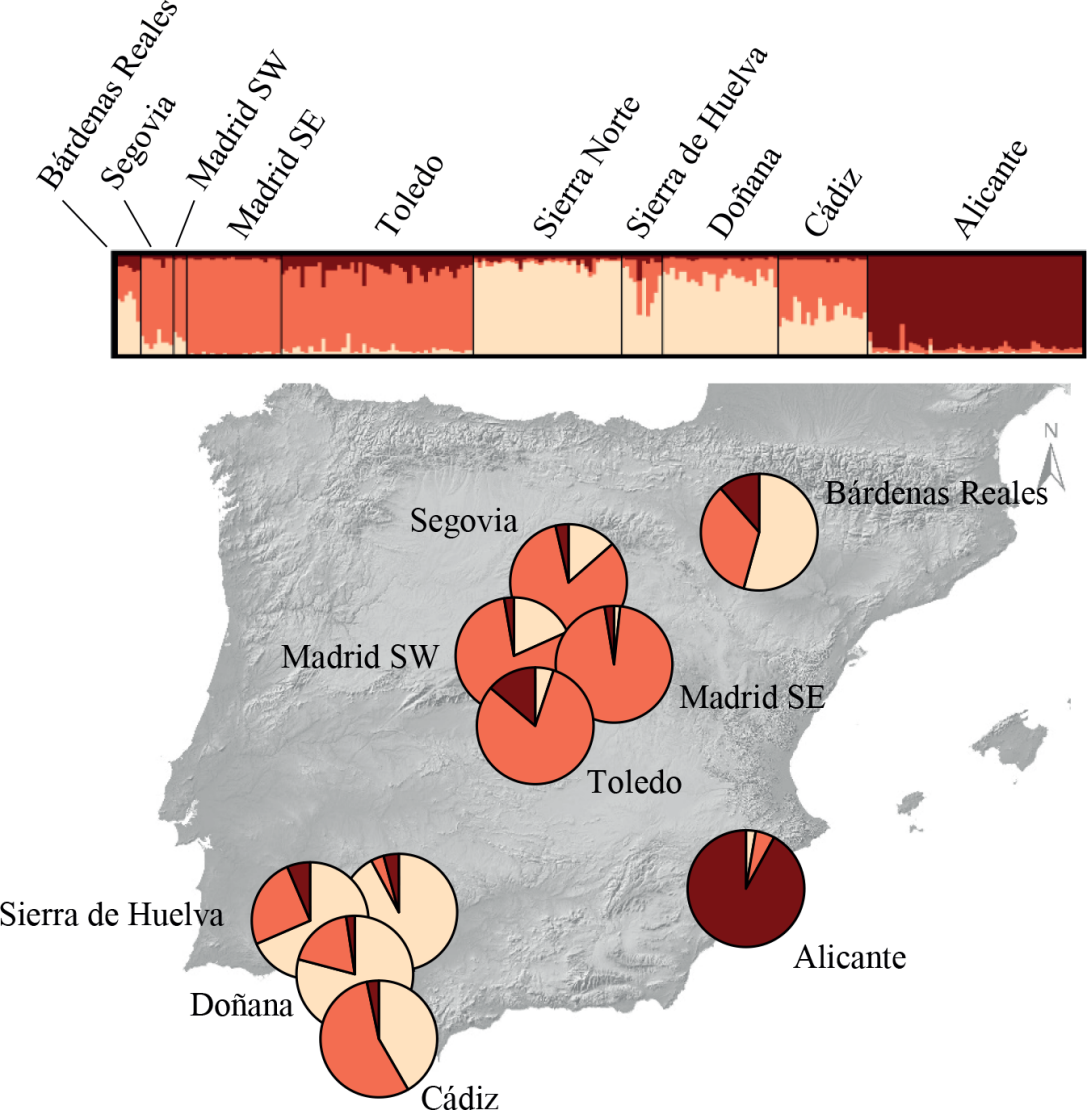
Genetic assignment of individuals based on the Bayesian method implemented in the program Structure. The number of inferred clusters was fixed to three, as this represents the most supported value of *K*. The admixture proportions generated by the software were represented using pie charts for each studied subpopulation, with each colour indicating a different genotypic cluster. Each individual is represented by a thin vertical bar, which is partitioned into coloured segments that represent the individual’s probability of belonging to the cluster with that colour.

**Fig 3:**
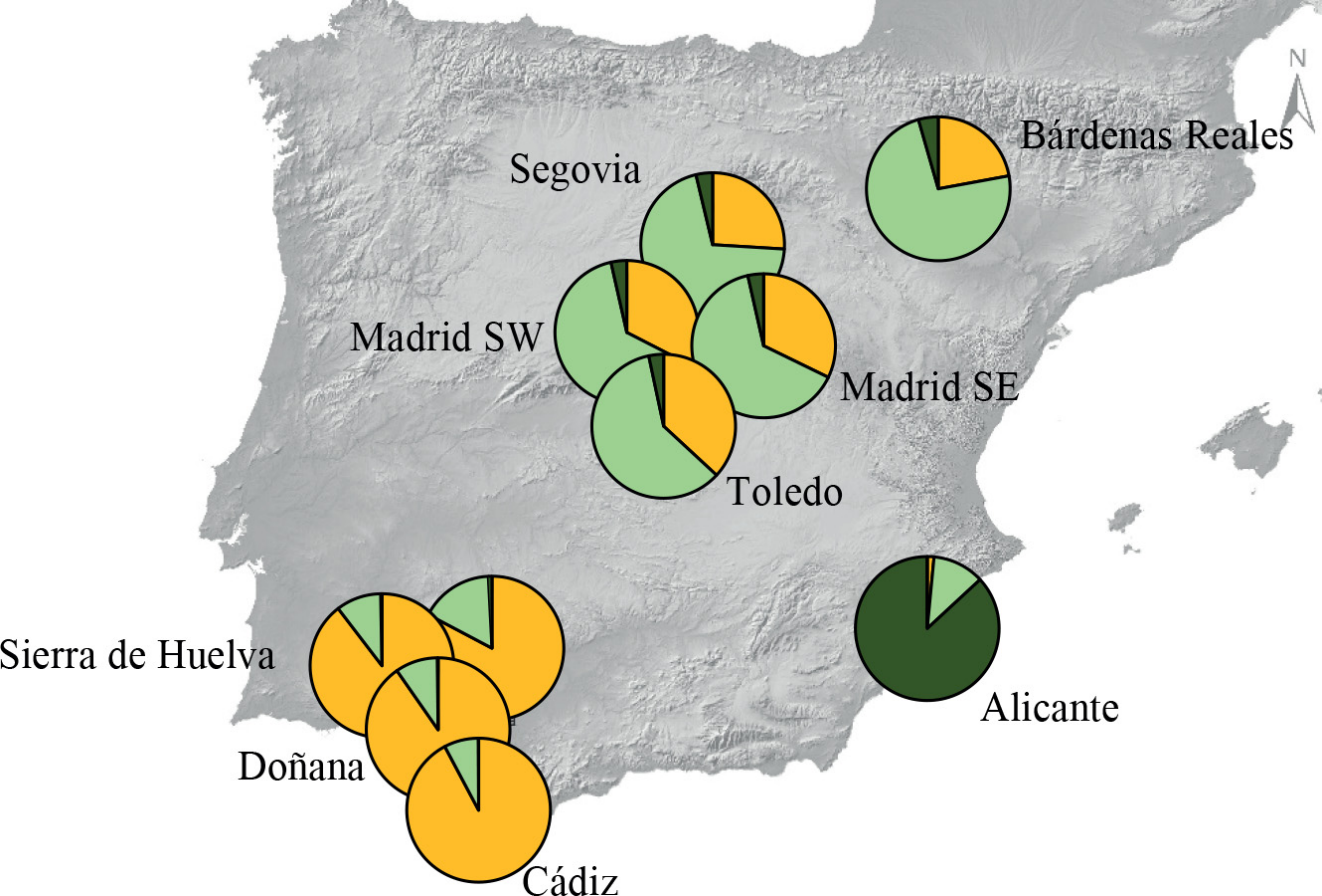
Spatial clustering analysis in Geneland. The number of inferred clusters was fixed to three, as this represents the most supported value of *K*. The admixture proportions for each studied subpopulation were represented using pie charts, with each colour indicating a different genotypic cluster.

Structure analyses without considering prior population information indicated that the number of genetic clusters best fitting the data set was *K* = 1 (i.e. LnPr(X|*K*) reached a maximum value for *K* = 1; S1 Fig). When the locality of origin was used as prior information, *K* = 3 was the most supported cluster solution (S1 Fig), splitting with moderate levels of admixture the subpopulations from Central Spain (Toledo, SW and SE Madrid and Segovia), south-western Spain (Sierra Norte, Sierra de Huelva and Doñana) and the single location at south-eastern Spain (Alicante, Fig 2). Individuals from Cádiz and Bardenas Reales showed the highest degree of admixed ancestries. Geneland offered similar results, with three also the most supported value of *K*. The next five posterior runs with the number of populations fixed to three showed good consistency and basically depicted the same pattern of differentiation, but including in the central cluster the samples from Bardenas Reales and in the south-western cluster the samples from Cádiz (Fig 3).

For the eight populations with samples sizes higher than six individuals, all pairwise *F*_ST_ estimates were significant (*p* < 0.05; S3 Table). The weakest differentiation (0.027) was found between Toledo and Segovia, two close localities from Central Spain, while the highest level of differentiation (0.112) was found between the furthest sites Sierra de Huelva and Alicante, located in the south-western and south-eastern of the Iberian Peninsula, respectively. *F*’_ST_ values offered similar results, with Toledo and Segovia the sites with the lowest differentiation (0.096), but in this case the distant Sierra de Huelva and Madrid SE showed the highest differentiation (0.387). Moreover, both statistics showed a significant correlation with the geographical distance between sites (Mantel tests, *F*_ST_: *r* = 0.361, *p* = 0.03; *F*’_ST_: *r* = 0.393, *p* = 0.03).

Despite the low levels of genetic differentiation found among sampling sites, AMOVA analyses statistically supported the reported substructure and maximized the differentiation among the inferred clusters when Cádiz was assigned to the south-western cluster, revealing that 1.84% of the global variation is found among clusters, 4.39% among sampling sites within these three groups, 16.04% among individuals within sites and the remaining 77.73% corresponds to individual variation (*F*_CT_ = 0.02; *F*_SC_ = 0.05; *F*_IS_ = 0.17; *F*_IT_ = 0.22; all *p* = 0.001). Considering these groups in a one-way ANOVA, we did not find significant differences in descriptive statistics of genetic diversity among them (all *p* > 0.05; Table 1). In general, subpopulations showed moderate levels of genetic diversity (Table 1) and the estimates of effective population size resulted similar and relatively high for all the groups (South-eastern Spain: *Ne* = 225.4, 95% CI = 107.2–2376.1; South-western Spain: *Ne* = 200.5, 95% CI = 94.7–2329.0; Central Spain: *Ne* = 263.5, 95% CI = 129.0–1878.2).

**Table 1:** Statistical descriptors of genetic diversity for subpopulations and inferred genetic groups (in bold).

Inferred genetic group	Subpopulations	N	*N*_A_	*A*_E_	*H*_O_	u*H*_E_	*F*_IS_
**South-eastern Spain**	Alicante	**52**	**7.75**	**3.74**	**0.64**	**0.68**	**0.04**
**South-western Spain**		**96**	**9.13**	**4.86**	**0.62**	**0.72**	**0.13**
	Cádiz	22	5.88	3.92	0.67	0.71	0.02
	Doñana	28	7.13	4.09	0.68	0.71	0.03
	Sierra de Huelva	10	4.38	3.32	0.57	0.72	0.12
	Sierra Norte	36	7.50	4.20	0.57	0.68	0.17
**Central Spain**		**78**	**8.38**	**4.08**	**0.64**	**0.71**	**0.10**
	Segovia	8	5.25	3.63	0.72	0.73	-0.08
	Toledo	47	7.13	3.70	0.65	0.70	0.08
	Madrid SE	23	6.75	4.03	0.62	0.70	0.10
Not assigned to any group							
	Madrid SW	3	3.13	2.77	0.65	0.68	-0.15
	Bardenas Reales	6	4.50	3.42	0.73	0.70	-0.16

Each studied subpopulation was assigned to a genetic group using Bayesian clustering methods and AMOVAs analyses (groups in bold). Madrid SW and Bardenas Reales were not included in any group due to their low sample size and the presence of siblings in the sample, respectively.

N, number of genotyped individuals; *N*_A_, mean number of alleles; *A*_E_, mean number of effective alleles; *H*_O_, mean observed heterozygosity; u*H*_E_, mean unbiased expected heterozygosity; *F*_IS_, mean inbreeding coefficient. ANOVA tests for these descriptors yielded non-significant differences between subpopulations grouped by the inferred genetic groups: (*N*_A_) *F*_1,5_ = 0.6; *p* = 0.58; (*N*_E_) *F*_1,5_ = 0.11; *p* = 0.9; (*H*_O_) *F*_1,5_ = 0.44; *p* = 0.67; (u*H*_E_) *F*_1,5_ = 1.15; *p* = 0.39; (*F*_IS_) *F*_1,5_ = 0.36; *p* = 0.72

### Inference of past demographic processes

Heterozygosity tests did not reveal signatures of heterozygosity excess and, in consequence, we did not infer recent demographic bottlenecks at any of the three genetic groups most supported by AMOVA analyses. Contrarily, we found a significant defect of heterozygosity in some of the analyses, which may reflect the recent demographic expansion of the species in some places. However, the results of the analyses notably varied depending of the mutation model, with the SMM model being more prone to yield significant signatures of expansion (Table 2). Moreover, five out of the six Bottleneck tests yielded similar results when loci that showed a significant homozygote excess were excluded from the analyses (Table 2).

**Table 2:** Inference of past demographic processes at the inferred genetic groups using software.

	Wilcoxon test					
		All loci		Loci in HWE		
Inferred genetic group	Mutation model	Het. excess	Het. deficiency	Het. excess	Het. deficiency	Discarded loci
South-eastern Spain (*n* = 52; Alicante)	TPM	0.99	**0.02**	0.96	**0.05**	Oe2-57, Oe054
	SMM	0.99	**0.01**	0.98	**0.04**	Oe2-57, Oe054
South-western Spain (*n* = 96; Cádiz, Doñana, Sierra Norte, Sierra de Huelva)	TPM	0.73	0.32	0.77	0.29	Oe2-57
	SMM	0.98	**0.03**	0.97	**0.04**	Oe2-57
Central Spain (*n* = 78; Segovia, Toledo, Madrid, SE)	TPM	0.62	0.42	0.66	0.42	Oe054, Bb101
	SMM	0.97	**0.03**	0.78	0.28	Oe054, Bb101

Analyses were carried using one-tailed Wilcoxon’s tests under the stepwise mutation model (SMM) and the two-phase mutation model (TPM; variance = 3.1, proportion of multi-step mutation = 22%). Each analysis was repeated discarding those loci that showed a significant homozygote excess after Bonferroni correction (*p* < 0.05). Significant and marginally significant values in bold.

**Fig 4:**
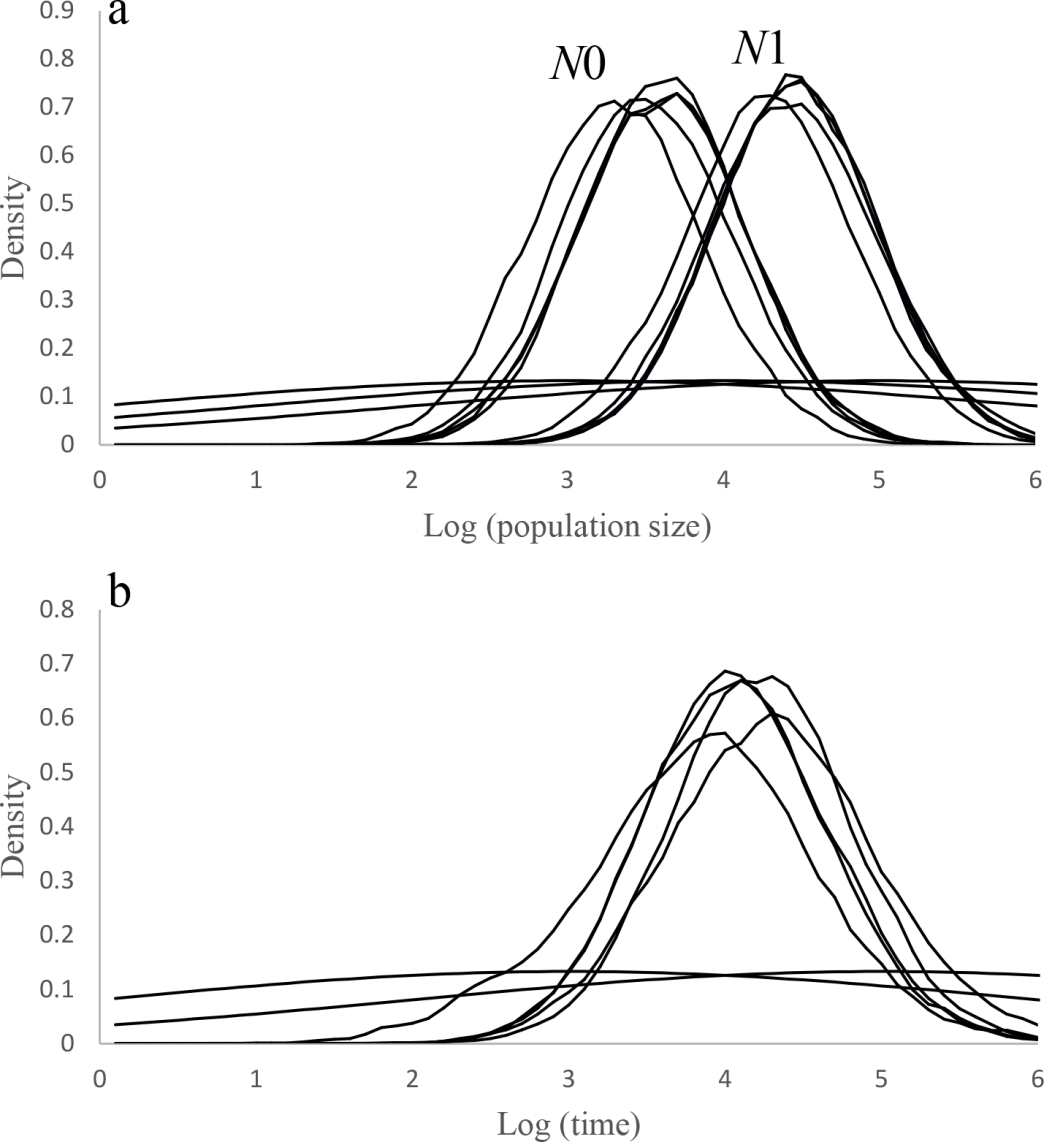
Inference of the demographic history of the Eurasian eagle owl (*Bubo bubo*) in Spain. Results obtained from the analyses with Msvar 1.3. Solid lines correspond to the posterior distribution of the parameters obtained in the five different runs. The wide uninformative priors employed in the analyses are represented by the dashed lines. (a) Posterior distribution of ancestral (N1) and present (N0) population effective sizes. (b) Posterior distribution of the time (in years ago) since the population collapse.

Despite the different values of θ assumed, M-ratio analyses did not show significant bottleneck signals at any of the three inferred groups (Table 3). However, Msvar 1.3 reflected insights of an ancient population collapse at the whole population, which was dated approximately 10,000 years ago (10th–90th quantiles = 1,584–79,432). The effective population *N*1 decreased over 10 times from a larger ancestral effective size estimated around 25,119 individuals (10th–90th quantiles = 5,012–125,893), to the current effective size *N*0 of 3,162 (10th–90th quantiles = 631–15,849). The obtained posterior densities of *N*0, *N*1 and *T* slightly varied over the different runs and the different priors used, depicting in all cases a population decline of one order of magnitude in the effective population size dated thousands of years ago (Fig 4, S4 Table).

**Table 3:** Parameters and results of the M-ratio analyses used to detect significant reductions in the effective population sizes of the inferred clusters.

	M-ratio			
Inferred genetic group	*Ne*	θ	M	M_c_
South-eastern Spain (*n* = 52; Alicante)	5	0.01	0.84	0.77
	500	1	0.84	0.7
	1000	2	0.84	0.68
	3000	6	0.84	0.67
	6000	12	0.84	0.67
	25000	50	0.84	0.63
South-western Spain (*n* = 96; Cádiz, Doñana, Sierra Norte, Sierra de Huelva)	5	0.01	0.84	0.76
	500	1	0.84	0.7
	1000	2	0.84	0.7
	3000	6	0.84	0.7
	6000	12	0.84	0.7
	25000	50	0.84	0.69
Central Spain (*n* = 78; Segovia, Toledo, Madrid SE)	5	0.01	0.9	0.77
	500	1	0.9	0.7
	1000	2	0.9	0.69
	3000	6	0.9	0.69
	6000	12	0.9	0.69
	25000	50	0.9	0.67

We did not find significant evidences of recent bottlenecks at any of the inferred groups using Bayesian clustering methods and AMOVAs analyses *Ne*, assumed pre-bottleneck effective population size; θ, calculated as θ = 4*Ne*μ, considering a mutation rate (μ) of 5x10^-4^; M, calculated as M = *k*/*r*, where *k* is the number of alleles and *r* is the range of allele sizes; M_C_, critical value set at the lower 5% tail of the distribution below which it can be assumed that the population has experienced a significant reduction in size.

## Discussion

### Current spatial patterns of genetic structure

In this study we found some genetic signatures which could be in part attributable to the recent population dynamics experienced by eagle owls in the Iberian Peninsula, but also probably related with the species’ past demographic history and traits. Concretely, we found a weak but consistent pattern of genetic structure revealed by both Bayesian clustering and AMOVAs analyses, and a significant correlation between genetic and geographical distances. In the Iberian Peninsula, spatial genetic patterns like this have been reported for other long-lived raptor species that also experienced recent population declines as the Spanish imperial eagle (*Aquila adalberti*) [50, 51] and the Bonelli’s eagle (*Aquila fasciata*) [52]. Such spatial patterns of differentiation could be thought as a direct consequence of the isolation of the species in different areas and its posterior spatial connection due to the species’ expansion [53, 54]. However, in this geographic context and for recovering species with high dispersal capacity, the presence of discrete genetic units is likely to be ephemeral, decreasing the differentiation among clusters with time since the expansion [55]. Instead, some factors such as ongoing anthropogenic pressures or philopatry could be delaying or impeding the homogenization of the genetic signal left by past population fragmentation [50, 51, 56, 57].

### Demographic history of the Iberian population

Demographic analyses failed to detect genetic signatures of recent bottlenecks in the inferred groups, which may indicate that past population declines were not severe enough to leave detectable signals on the species genetic makeup. Thus, a possibility is that the decline of eagle owl populations in the recent past has not reduced effective population sizes below the threshold that is necessary to produce genetic signals associated with such demographic events. Alternatively, the recovery and range expansion of the species over the last 40 years could have eroded some of the genetic signatures associated with local population declines. Unlike most large raptors such as eagles or vultures, the eagle owl has high reproductive rates and this may have favored a rapid demographic recovery [58]. Further, the breeding performance of some of the studied populations of eagle owl is among the highest reported for the species across its distribution range [59–64]. Accordingly, Bottleneck analyses detected population expansions in some of the inferred groups, a pattern that was even consistent when excluding loci that deviate from HWE. These findings were also supported by the moderated levels of genetic diversity and the large effective population sizes estimated for the studied populations. However, it remains unclear whether subsequent demographic changes can erase the genetic signatures of recent demographic bottlenecks, which is likely to depend among other things on the population growth rate and the impact of immigration and admixture with individuals from other populations during the expansion phase [65, 66]. Study systems like this, with known and complex demographic histories, are valuable to explore the limits of the current methodological approaches. In particular, we found discrepancies in the detection of expansions depending on the mutation model assumed in Bottleneck analyses. SMM showed a higher power, despite for most microsatellite loci TPM is thought to be more adequate [45].

At the whole population level, Msvar analyses found signatures of an ancient population collapse that started after the end of the last glacial period. Both this and previous studies have generally found that the Storz and Beaumont method [49] shows a preponderance to detect old and severe bottlenecks in comparison with M-ratio and Bottleneck analyses [67]. Accordingly, several previous studies attempted to address the demographic effects of recent anthropogenic disturbances on populations by using this approach also found ancient population collapses linked to past climate or habitat changes, as occurred for giant pandas in China (*Ailuropoda melanoleuca*) [68], fishers in California (*Martes pennanti*) [69] or European white elms (*Ulmus laevis*) [70] and Iberian lynxes in Spain [71]. Although the severity of ancient bottlenecks and its temporal estimation could be biased by the population genetic structure, the sampling scheme or the current gene flow with other populations, and therefore should be interpreted with caution [72]. Overall our results support previous studies with empirical and simulated data finding that bottleneck detection methods generally perform poorly at identifying very recent or weak population declines [65, 66, 73, 74].

### Genetic patterns and dispersal

**Fig 5:**
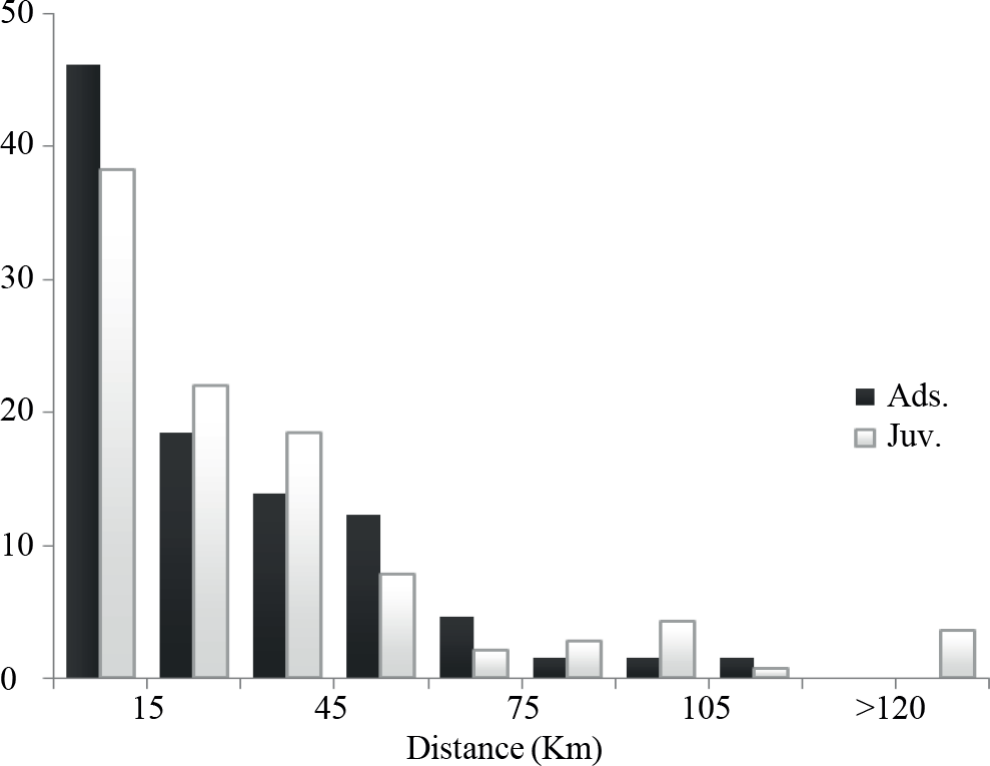
Eagle owl distance recoveries in Spain during the period 1973–2010. No differences were detected between bird ringed as chicks or juveniles (Euring 1 and 3) and adults (Pearson's Chi-squared test, χ_8_^2^ = 7.31, *p* = 0.503, *n* = 206).

Although eagle owls can potentially disperse large distances, our results agree with recovery data of banded individuals and evidence of limited dispersal among subpopulations (Fig 5), supporting the hypothesis of population fragmentation by human pressures as it has been described for other endangered raptors in the Iberian Peninsula [50–52]. Accordingly, a previous study on eagle owls conducted at a local scale in southeastern Spain found genetic structuring due to limited gene flow among close demes [75]. These authors concluded that landscape structure and geographical isolation were determinant at small spatial scales (10–100 km^2^). In a wider geographical context, one of the most important anthropogenic negative impacts on population connectivity are probably electrocutions. Electrocution became the major source of non-natural mortality of eagle owls after the legal protection of raptors in Spain [13, 76, 77]. Eagle owl high density areas often match with agroecosystems that sustain large populations of rabbits but also are often crossed by power lines, a fact that might have promoted high population turnover rates in low quality breeding areas [60]. In dense populations with high breeding success, frequent mortality might create gaps in the breeding population, which could be quickly occupied by floaters [62]. This effect might lead to territorial packing, increasing density but also limiting dispersal rates among populations [78, 79].

Besides, some natural factors might have also increased population fragmentation. Eagle owl traits such as philopatry, the tendency to breed close to birthplaces [80, 81], or conspecific attraction, the tendency to settle close to conspecifics, have been previously reported as important forces limiting realized dispersal [82]. Moreover, habitat selection linked to rabbit population dynamics could have also fragmented owl populations since the outbreak of rabbit viral diseases in the 1950s. These disease outbreaks led to a global decline in rabbit populations that resulted in local extinctions and had profound effects on the diet, breeding performance and survival of many predators in Spain, including eagle owls [83, 84]. The naturally patchy distribution of suitable habitats of rabbits together with the geographically patchy pattern of recovery in this species, has led to high and low prey density areas that might have further contributed to shape a fragmented distribution of eagle owl populations [85, 86].

## Conclusions

Overall, our analyses indicate that population declines were not severe enough to leave detectable genetic signals or that such signals have been eroded by the rapid demographic recovery experienced by the species in recent years. The combination of limited dispersal and high turnover and reproductive rates might have contributed to both the observed patterns of spatial genetic structure and the short-term erosion of the genetic signatures of recent demographic bottlenecks in Iberian eagle owls. In fact, we found moderate levels of genetic diversity and no differences in descriptive statistics of genetic diversity between the inferred clusters. Somewhat lesser levels of genetic variation were reported for the Scandinavian population of eagle owls, which passed a bottleneck below a few hundreds of individuals (*H*_E_ = 0.61; [29]); and a similar pattern of genetic differentiation was reported for the endangered Blakiston's fish owl (*Bubo blakistoni*) on Hokkaido Island in Japan, a population with a significant decline of allelic richness over the last three decades [87]. In our case, further studies considering populations with stable demographic histories or samples coming from museum collections could help to elucidate if past demographic declines have negatively impacted the levels of genetic diversity of Iberian eagle owl populations. Genetic structure is expected to have a time lag in its response to changes in gene flow, which may explain the contemporary patterns of genetic structure observed despite the species demographic recovery and expansion [88].

## Supporting Information

S1 Fig Results of Bayesian clustering analyses in Structure.
(a) Without considering prior population information and (b) using population identity as prior information (“Locprior” option). Plots show the mean (± SD) log probability of the data (ln Pr(X|*K*)) (left axis, black dots and error bars) and the magnitude of Δ*K* as a function of *K* (right axis, open dots) over 11 runs.
(DOCX)

S1 Table Characteristics and variability of microsatellite loci analysed for the studied subpopulations of eagle owl (*Bubo bubo*). For labelled primers the dye is reported within brackets. T_a_, annealing temperature; *N*_A_, number of alleles; u*H*_E_, unbiased expected heterozygosity; *H*_O_, observed heterozygosity. *Significant heterozygote deficiency after Bonferroni correction (*p* < 0.05).
(DOCX)

S2 Table Parameters for Msvar 1.3 simulations. In bold those values changing from the previous simulation.
(DOCX)

S3 Table Pair-wise estimates of genetic differentiation. *F*_ST_ values (below diagonal) and *F*’_ST_ values (above diagonal).
(DOCX)

S4 Table Past demographic inference of the eagle owl population from Spain using Msvar 1.3. N0 and N1 are the current and ancestral effective population sizes respectively. Time (*T*) represents the date of the change in population size from *N*0 to *N*1.
(DOCX)
