# Supplementary material for: Genetic Signatures of Demographic Changes in an Avian Top Predator during the Last Century: Bottlenecks and Expansions of the Eurasian Eagle Owl in the Iberian Peninsula
Source: PLoS One. 2015 Jul 31;10(7):e0133954. doi: 10.1371/journal.pone.0133954 (PMC4521928; doi:10.1371/journal.pone.0133954)
Supplement: S1 Table — For labelled primers the dye is reported within brackets. Ta, annealing temperature; N A, number of alleles; uH E, unbiased expected heterozygosity; H O, observed heterozygosity. *Significant heterozygote deficiency after Bonferroni correction (p < 0.05). (DOCX) [file pone.0133954.s002.docx]

| Locus | Repeat motif | Primer sequence | T_a_ (ºC) | Size range (bp) | N_A_ | uH_E_/H_O_ |
| --- | --- | --- | --- | --- | --- | --- |
| Bb126 | (GA)_n_ | F_(6-FAM)_-TCTCCAGAAGGGTTGTCATC | 45 | 196-218 | 8 | 0.74/0.69 |
|  |  | R-TGCTAAAACCTTACAGAATAACAG |  |  |  |  |
| Bb101 | (AC)_n_ | F_(VIC)_-AATAACCCCAATAGAAGC | 45 | 172-178 | 4 | 0.36/0.33 |
|  |  | R-ACCAGAAGGAGATGAGACC |  |  |  |  |
| 15A6 | (GATA)_n_ | F_(PET)_-ACCTCAGAAGCAGACAGAACC | 50 | 111-165 | 14 | 0.87/0.81* |
|  |  | R-CCTTTCGCATTGCTGTAAC |  |  |  |  |
| Oe2-57 | (GAAA)_n_ | F_(NED)_-TTTATCTGAGTGGAAGGGTAGTGC | 50 | 269-347 | 24 | 0.89/0.65* |
|  |  | R-CCAACTAAACACTATCTTTCTCC |  |  |  |  |
| Oe3-7 | (GATA)_n_ | F_(VIC)_-GTGGGTTTATTGCCCCCTCG | 50 | 105-137 | 9 | 0.80/0.79 |
|  |  | R-CAGATGAATTGAATGGATAGATGG |  |  |  |  |
| Oe045 | (GATA)_n_GATTA(GATA)_n_ | F_(NED)_-GTATGTTCTACGTTTGGATTTCCA | 50 | 157-207 | 15 | 0.83/0.74* |
|  |  | R-AAACCTGGCAAGTGCTGTT |  |  |  |  |
| Oe054 | (GATA)_n_ | F_(6-FAM)_-TCAGAAAGAAAACTTCAGCAACC | 50 | 78-112 | 9 | 0.65/0.51* |
|  |  | R-CATATATGTATACACAGGCACATGC |  |  |  |  |
| Oe128 | (GATA)_n_ | F_(VIC)_-CGTTGTAAATGATGAATCGCCTAGTGC | 50 | 277-305 | 8 | 0.65/0.57* |
|  |  | R-ATGCATGTATACATACAAACCTGG |  |  |  |  |
