# Supplementary material for: Genetic Signatures of Demographic Changes in an Avian Top Predator during the Last Century: Bottlenecks and Expansions of the Eurasian Eagle Owl in the Iberian Peninsula
Source: PLoS One. 2015 Jul 31;10(7):e0133954. doi: 10.1371/journal.pone.0133954 (PMC4521928; doi:10.1371/journal.pone.0133954)
Supplement: S2 Table — In bold those values changing from the previous simulation. (DOCX) [file pone.0133954.s003.docx]

|  | Starting parameters of priors | | | | | | | | Hyperpriors | | | | | | | | | | | | | | | |
| --- | --- | --- | --- | --- | --- | --- | --- | --- | --- | --- | --- | --- | --- | --- | --- | --- | --- | --- | --- | --- | --- | --- | --- | --- |
|  | log(*N_0_*) | | log(*N_1_*) | | log(*θ*) | | log(T) | | log(*N_0_*) | | | | log(*N_1_*) | | | | log(*θ*) | | | | log(*T*) | | | |
| Run 1 | 4 | 1 | 4 | 1 | -3.5 | 1 | 5 | 1 | 4 | 3 | 0 | 0.5 | 4 | 3 | 0 | 0.5 | -3.5 | 0.5 | 0 | 2 | 5 | 3 | 0 | 0.5 |
| Run 2 | 4 | 1 | 4 | 1 | -3.5 | 1 | 5 | 1 | **5** | 3 | 0 | 0.5 | 4 | 3 | 0 | 0.5 | -3.5 | 0.5 | 0 | 2 | 5 | 3 | 0 | 0.5 |
| Run 3 | 4 | 1 | 4 | 1 | -3.5 | 1 | 5 | 1 | **3** | 3 | 0 | 0.5 | 4 | 3 | 0 | 0.5 | -3.5 | 0.5 | 0 | 2 | 5 | 3 | 0 | 0.5 |
| Run 4 | 4 | 1 | 4 | 1 | -3.5 | 1 | 5 | 1 | 4 | 3 | 0 | 0.5 | 4 | 3 | 0 | 0.5 | -3.5 | 0.5 | 0 | 2 | **3** | 3 | 0 | 0.5 |
| Run 5 | 4 | 1 | 4 | 1 | -3.5 | 1 | **2** | 1 | 4 | 3 | 0 | 0.5 | 4 | 3 | 0 | 0.5 | -3.5 | 0.5 | 0 | 2 | 3 | 3 | 0 | 0.5 |
