# Supplementary material for: Genetic Signatures of Demographic Changes in an Avian Top Predator during the Last Century: Bottlenecks and Expansions of the Eurasian Eagle Owl in the Iberian Peninsula
Source: PLoS One. 2015 Jul 31;10(7):e0133954. doi: 10.1371/journal.pone.0133954 (PMC4521928; doi:10.1371/journal.pone.0133954)
Supplement: S3 Table — F ST values (below diagonal) and F’ST values (above diagonal). (DOCX) [file pone.0133954.s004.docx]

|  | Alicante | Cádiz | Doñana | Sierra de Huelva | Sierra Norte | Madrid SE | Segovia | Toledo |
| --- | --- | --- | --- | --- | --- | --- | --- | --- |
| Alicante | - | 0.262 | 0.232 | 0.366 | 0.129 | 0.192 | 0.158 | 0.139 |
| Cádiz | 0.081 | - | 0.177 | 0.142 | 0.200 | 0.319 | 0.252 | 0.308 |
| Doñana | 0.066 | 0.049 | - | 0.113 | 0.148 | 0.193 | 0.139 | 0.233 |
| Sierra de Huelva | 0.112 | 0.042 | 0.030 | - | 0.311 | 0.387 | 0.254 | 0.355 |
| Sierra Norte | 0.040 | 0.061 | 0.041 | 0.093 | - | 0.197 | 0.119 | 0.188 |
| Madrid SE | 0.057 | 0.093 | 0.051 | 0.110 | 0.058 | - | 0.122 | 0.100 |
| Segovia | 0.046 | 0.071 | 0.035 | 0.069 | 0.034 | 0.032 | - | 0.096 |
| Toledo | 0.042 | 0.093 | 0.065 | 0.105 | 0.057 | 0.029 | 0.027 | - |
