# Supplementary material for: Genetic Signatures of Demographic Changes in an Avian Top Predator during the Last Century: Bottlenecks and Expansions of the Eurasian Eagle Owl in the Iberian Peninsula
Source: PLoS One. 2015 Jul 31;10(7):e0133954. doi: 10.1371/journal.pone.0133954 (PMC4521928; doi:10.1371/journal.pone.0133954)
Supplement: S4 Table — N0 and N1 are the current and ancestral effective population sizes respectively. Time (T) represents the date of the change in population size from N0 to N1. (DOCX) [file pone.0133954.s005.docx]

| **Run** | **log(*N*0) 50% (10-90%)** | **log(*N*1) 50% (10-90%)** | **log(*T*) 50% (10-90%)** |
| --- | --- | --- | --- |
| 1 | 3.5 (2.8-4.1) | 4.4 (3.7-5.1) | 4 (3.3-4.8) |
| 2 | 3.6 (2.9-4.3) | 4.4 (3.7-5.1) | 4 (3.3-4.8) |
| 3 | 3.3 (2.5-4) | 4.4 (3.7-5.1) | 3.8 (2.8-4.7) |
| 4 | 3.6 (2.8-4.3) | 4.2 (3.5-5) | 4.2 (3.4-4.9) |
| 5 | 3.6 (2.9-4.2) | 4.4 (3.7-5.1) | 3.2 (3.4-5.1) |
