# Supplementary material for: Genetic Signatures of Demographic Changes in an Avian Top Predator during the Last Century: Bottlenecks and Expansions of the Eurasian Eagle Owl in the Iberian Peninsula
Source: PLoS One. 2015 Jul 31;10(7):e0133954. doi: 10.1371/journal.pone.0133954 (PMC4521928; doi:10.1371/journal.pone.0133954)
Supplement: Supplementary file 5 [file pone.0133954.s001.docx]

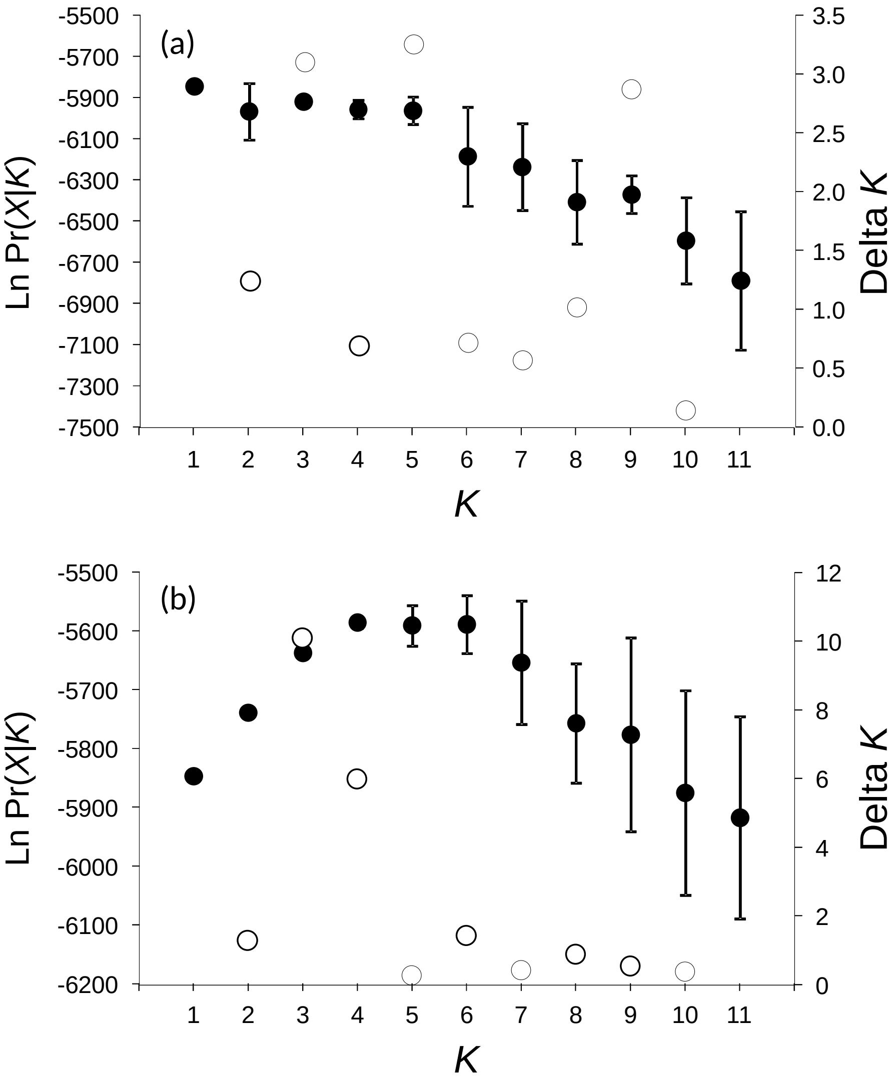
(a)
-5500
-5700
-5900
-6100
-6300
-6500
-6700
-6900
-7100
-7300
-7500
Ln Pr(X|K)
3.5
3.0
2.5
2.0
1.5
1.0
0.5
0.0
Delta K
1 2 3 4 5 6 7 8 9 10 11
K
(b)
-5500
-5600
-5700
-5800
-5900
-6000
-6100
-6200
Ln Pr(X|K)
12
10
8
6
4
2
0
Delta K
1 2 3 4 5 6 7 8 9 10 11
K
